# Trends and regional distribution of outpatient claims for asthma, 2009–2016, Germany

**DOI:** 10.2471/BLT.19.229773

**Published:** 2019-11-01

**Authors:** Manas K Akmatov, Jakob Holstiege, Annika Steffen, Jörg Bätzing

**Affiliations:** a Department of Regional Health Care Analysis and Health Care Atlas, Central Research Institute of Ambulatory Health Care in Germany, Salzufer 8, 10587, Berlin, Germany.

## Abstract

**Objective:**

To investigate asthma morbidity in Germany by calculating current prevalence, examining its temporal and spatial trends and estimating the total number of asthmatics in Germany and calculating age-, sex- and residence-specific risk.

**Methods:**

We used claims data reported by physicians during 2009–2016, including outpatient diagnoses of all statutory health insured individuals, comprising 85.3% (70 416 019/82 521 653) of the total population in Germany in 2016. We performed a spatial analysis of asthma prevalence according to administrative district by calculating Global and Local Moran’s *I*. We assessed the risk of asthma by sex, age, type of residence (rural versus urban) and federal state (East versus West) using a multilevel parametric survival regression.

**Findings:**

We estimated that 4.7 million individuals were affected by asthma in 2016, including 0.8 million children and 3.9 million adults. We observed a slightly higher prevalence (with an increasing trend) among adults (5.85%; 3 408 622/58 246 299) compared to children (5.13%; 624 899/12 169 720), and calculated an age-standardized prevalence of 5.76% (95% confidence interval, CI: 5.76–5.77). We found evidence of a strong spatial autocorrelation (Global Moran’s *I*: 0.50, *P* < 0.0001), and identified local spatial clusters with higher levels of prevalence. Living in the western (versus eastern) federal states and living in densely populated large urban municipalities (versus rural area) were independently associated with an increased risk of asthma, with hazard ratios of 1.33 (95% CI: 1.32–1.34) and 1.32 (95% CI: 1.31–1.32), respectively.

**Conclusion:**

Our insights into the spatial distribution of asthma morbidity may inform public health interventions, including region-specific prevention programmes and control.

## Résumé

**Objectif:**

Étudier la morbidité de l’asthme en Allemagne en calculant la prévalence, en examinant les tendances temporelles et spatiales, en estimant le nombre total d’asthmatiques en Allemagne et en calculant le risque par âge, sexe et lieu de résidence.

**Méthodes:**

Nous avons utilisé les demandes de remboursement déclarées par les médecins pendant la période 2009–2016, notamment les diagnostics ambulatoires de toutes les personnes affiliées au régime d’assurance maladie obligatoire, représentant 85,3% (70 416 019/82 521 653) de la population allemande totale en 2016. Nous avons réalisé une analyse spatiale de la prévalence de l’asthme par district administratif en calculant l'indice de Moran global et local. Nous avons évalué le risque d’asthme par sexe, par âge, par type de résidence (rural contre urbain) et par État fédéré (Est contre Ouest) à l’aide d’une régression de survie paramétrique multiniveaux.

**Résultats:**

Nous avons estimé que 4,7 millions de personnes souffraient d’asthme en 2016, dont 800 000 enfants et 3,9 millions d’adultes. Nous avons observé une prévalence légèrement plus élevée (avec une tendance à la hausse) chez les adultes (5,85%; 3 408 622/58 246 299) que chez les enfants (5,13%; 624 899/12 169 720), et nous avons calculé une prévalence standardisée sur l’âge de 5,76% (intervalle de confiance, IC, de 95%: 5,76–5,77). Nous avons constaté des signes d'une forte autocorrélation spatiale (indice de Moran global: 0,50, *P* < 0,0001), et identifié des grappes spatiales locales présentant une prévalence plus élevée. Le fait de vivre dans un État fédéré de l’Ouest (par rapport à l’Est) et dans une grande municipalité urbaine densément peuplée (par rapport à une zone rurale) est associé, de façon indépendante, à un risque accru d’asthme, avec un rapport de risque de respectivement 1,33 (IC 95%: 1,32–1,34) et 1,32 (IC 95%: 1,31–1,32).

**Conclusion:**

Ces informations sur la répartition spatiale de la morbidité de l’asthme peuvent éclairer les interventions de santé publique, notamment les programmes de prévention et de contrôle adaptés à chaque région.****

## Resumen

**Objetivo:**

Investigar la morbilidad por asma en Alemania mediante el cálculo de la prevalencia actual, el examen de sus tendencias temporales y espaciales, la estimación del número total de asmáticos en Alemania y el cálculo del riesgo específico por edad, sexo y residencia.

**Métodos:**

Se utilizaron los datos de reclamaciones notificados por los médicos durante 2009-2016, incluidos los diagnósticos de pacientes externos de todos los asegurados estatutarios de salud, que representan el 85,3 % (70 416 019/82 521 653) de la población total en Alemania en 2016. Se realizó un análisis espacial de la prevalencia del asma según el distrito administrativo calculando el I de Moran a nivel global y local. Se evaluó el riesgo de asma por sexo, edad, tipo de residencia (rural versus urbana) y estado federal (Este versus Oeste) mediante una regresión paramétrica de supervivencia multinivel.

**Resultados:**

Se estimó que 4, 7 millones de personas fueron afectadas por el asma en 2016, incluyendo 0,8 millones de niños y 3,9 millones de adultos. Se observó una prevalencia ligeramente mayor (con una tendencia creciente) entre los adultos (5,85 %; 3 408 622/58 246 299) en comparación con los niños (5,13 %; 624 899/12 169 720), y se calculó una prevalencia estandarizada por edad de 5,76 % (intervalo de confianza del 95 %, IC: 5,76 a 5,77). Se encontraron pruebas de una fuerte autocorrelación espacial (I de Moran Global: 0,50, *P* < 0,0001), y se identificaron los grupos espaciales locales con niveles más altos de prevalencia. Vivir en los estados federales occidentales (versus orientales) y vivir en municipios urbanos grandes densamente poblados (versus área rural) se asoció independientemente con un mayor riesgo de asma, con cocientes de riesgo de 1,33 (IC del 95 %: 1,32 a 1,34) y 1,32 (IC del 95 %: 1,31 a 1,32), respectivamente.

**Conclusión:**

Esta información sobre la distribución espacial de la morbilidad por asma puede servir de base para las intervenciones de salud pública, incluidos los programas de prevención y control específicos de cada región.

## ملخص

**الغرض:**

استقصاء معدلات الإصابة بالربو في ألمانيا عن طريق حساب معدل الانتشار الحالي، وفحص اتجاهاتها الوقتية والمكانية، وتقدير العدد الإجمالي لمرضى الربو في ألمانيا، وحساب المخاطر المتعلقة بالعمر والجنس والإقامة.

**الطريقة:**

لقد قمنا باستخدام بيانات المطالبات التي أبلغ عنها الأطباء خلال الفترة من 2009 إلى 2016، بما في ذلك التشخيصات في العيادات الخارجية لجميع الأفراد المؤمن عليهم بشكل قانوني، والتي تشمل 85.3% (70416019/82521653) من إجمالي عدد السكان في ألمانيا في عام 2016. كما قمنا بإجراء تحليلاً مكانياً لانتشار الربو وفقًا للمنطقة الإدارية من خلال الحساب بنظام موران العالمي والمحلي (Global and Local Moran). كذلك قمنا بتقييم خطر الإصابة بالربو حسب الجنس والعمر ونوع الإقامة (الريف مقابل الحضر)، والولاية الفيدرالية (الشرق مقابل الغرب)، باستخدام نموذج التحوف للبقاء الحدودي متعدد المستويات.

**النتائج:**

قم بتقدير أن 4.7 مليون شخص قد أصيبوا بالربو في عام 2016، بما يشمل 0.8 مليون طفل و3.9 مليون شخص بالغ.لاحظنا انتشاراً أعلى بقليل (مع اتجاه متزايد) بين البالغين (5.85%؛ 3408622/58246299) مقارنة بالأطفال (5.13%؛ 624899/12169720)، وقمنا بحساب معدل انتشار حسب العمر بنسبة 5.76% (فاصل الثقة 95%: 5.76 إلى 5.77). لقد وجدنا دليلًا على وجود ارتباط ذاتي مكاني قوي (جلوبال موران: 0.50، *نسبة احتمال *< 0.0001)، وحددنا مجموعات مكانية محلية ذات مستويات أعلى من الانتشار.أن العيش في كل من الولايات الفيدرالية الغربية (مقابل الشرقية)، والبلديات الحضرية الكبيرة كثيفة السكان (مقابل المناطق الريفية)، ارتبط بشكل مستقل بزيادة خطر الإصابة بالربو، حيث بلغت نسب المخاطرة 1.33 (فاصل الثقة 95%:1.32 إلى 1.34)، و1.32 (فاصل ثقة 95‏%:1.31 إلى 1.32)، على التوالي.

**الاستنتاج:**

إن رؤيتنا بخصوص التوزيع المكاني لمعدل الإصابة بالربو، قد تسهم في التعرف على التدخلات في الصحة العامة، بما في ذلك برامج الوقاية الخاصة بمنطقة ما والسيطرة عليها.

## 摘要

**目的:**

旨在通过计算当前患病率，检查其时间和空间趋势，估计德国哮喘患者的总数，并计算年龄、性别和特定居民风险，来调查德国哮喘发病率。

**方法:**

我们使用了 2009-2016 年期间由医生报告的索赔数据，包括所有依法参加医疗保险的个人【2016 年占德国总人口的 85.3%(70 416 019/82 521 653)】的门诊诊断。通过计算全球和当地的莫兰氏指数，按行政地区划分，对不同地区的哮喘患病率进行了空间分析。我们使用多层参数化回归模型，通过性别、年龄、居住类型（农村与城市）和联邦州（东部与西部）评估了哮喘的风险。

**结果:**

我们估计 2016 年有 470 万人患有哮喘，其中包括 80 万儿童和 390 万成人。我们观察到成年人 (5.85%; 3 408  622/58 246 299) 的患病率略高于儿童 (5.13%; 624 899/12 169 720)，并计算出年龄标准化患病率为 5.76%（95% 置信区间，CI：5.76–5.77）。我们发现了较强的空间自相关的证据（全球莫兰指数 ：0.50,*P* < 0.0001），并确定了具有较高患病率的局部空间集群。居住在西部（相对于东部）联邦州和人口稠密的大城市（相对于农村地区）与哮喘风险增加独立相关，风险比分别为 1.33（95% 置信区间：1.32-1.34 ）和 1.32（95% 置信区间：1.31-1.32）。

**结论:**

我们对哮喘发病率的空间分布的见解可能会为公共卫生干预提供信息，包括针对特定地区的预防方案和控制。****

## Резюме

**Цель:**

Изучить заболеваемость астмой в Германии посредством подсчета случаев заболевания в настоящее время, изучения таких факторов заболеваемости, как пространство и время, и оценки общего количества астматиков в Германии, а также расчета риска по возрасту, полу и месту проживания.

**Методы:**

Авторы использовали данные об амбулаторных диагнозах, поставленных врачами в течение 2009–2016 гг. всем лицам, имеющим государственную медицинскую страховку и составляющим 85,3% (70 416 019/82 521 653) от общей численности населения Германии в 2016 г. Авторы выполнили пространственный анализ распространенности случаев заболевания астмой по административным районам, рассчитав глобальный и локальный индекс Морана *I.* Была выполнена оценка риска развития астмы в зависимости от пола, возраста, места проживания (сельская местность или город) и федеральных земель (восток или запад) с использованием многоуровневой параметрической регрессионной модели выживаемости.

**Результаты:**

По оценке авторов, в 2016 году астмой болели 4,7 миллиона человек, в том числе 0,8 миллиона детей и 3,9 миллиона взрослых. Наблюдалась немного более высокая частота заболеваемости (с тенденцией к увеличению) среди взрослых (5,85%; 3 408 622/58 246 299) по сравнению с детьми (5,13%; 624 899/12 169 720). Была рассчитана стандартизированная по возрасту распространенность заболевания, составляющая 5,76% (с доверительным интервалом 95%, ДИ: 5,76–5,77). Авторы нашли подтверждение сильной пространственной автокорреляции (глобальный индекс Морана *I*: 0,50; *P* <0,0001) и выявили локальные пространственные кластеры с более высокой частотой заболеваемости. Проживание в западных (по сравнению с восточными) федеральных землях и в густонаселенных крупных городских муниципалитетах (по сравнению с сельской местностью) независимо ассоциировалось с повышенным риском развития астмы с коэффициентами риска 1,33 (95% ДИ: 1,32–1,34) и 1,32 (95% ДИ: 1,31–1,32) соответственно.

**Вывод:**

Понимание пространственного распределения показателя заболеваемости астмой может способствовать принятию надлежащих мер в области общественного здравоохранения, включая организацию программ по профилактике и контролю астмы в регионах с повышенной заболеваемостью этой болезнью.

## Introduction

Asthma is the most frequently diagnosed and chronic, noncommunicable, inflammatory disorder among children and adults. According to the latest Global Asthma Report, nearly 340 million individuals worldwide have been diagnosed with asthma;^1^ it is estimated that an additional 100 million individuals will be affected by 2025.^2^ The prevalence of asthma varies substantially across the globe, and has been shown to vary between countries by up to a factor of 21.^3^ Prevalence tends to be higher in developed countries, with the highest reported prevalence of asthma in Australia (21%), Sweden (20%), the United Kingdom of Great Britain and Northern Ireland (18%), the Netherlands (15%) and Brazil (13%); the lowest prevalence of asthma has been observed for Viet Nam (0.8%) and China (0.2%).^3^ Some studies, for example in Australia and the United States of America,^4^ United Kingdom^5^ and Latin American countries,^6^ have reported within-country variations. A higher degree of urbanization, associated with a higher exposure to risk factors (e.g. pollution or prenatal stress), has been linked to an increased risk of asthma.^7^^,^^8^ Another factor increasing this difference is the so-called hygiene hypothesis,^9^ which describes how growing up in a rural environment, with its associated increase in exposure to microbial agents^10^ and endotoxins,^11^ can have a protective effect against allergic diseases including asthma.

Prevalence estimates of 3–12% among children and 2–5% among adults have been reported in Germany;^12^ however, current estimates of asthma incidence are lacking. Regional variations in Germany have only been examined for rough geographical units and for specific age groups (e.g. children or adults). For example, one study demonstrated differences in asthma prevalence among children between East and West Germany.^13^ Another study involving only adult participants investigated variations in asthma prevalence across the German federal states.^14^

An examination of regional variation in asthma morbidity is of particular importance as geographical factors, and not just factors related to individual patients, play a considerable role in the pathogenesis of asthma.^10^ We therefore provide estimates of asthma morbidity in Germany for the years 2009 to 2016, and examine differences in prevalence with time, residence type (urban versus rural) and geographical location. We also estimate the total number of individuals in Germany currently affected by asthma, and calculate the sex-, age- and residence-specific risk of asthma incidence.

## Methods

### Data and study population

We used nationwide ambulatory claims data reported by physicians approved to treat statutory health insured individuals in Germany, acquired during 2009–2016. Privately insured members of the population were not included in this study. Claims data contain information on the sex, age and district of residence of outpatients (Germany’s 16 federal states included 402 administrative districts in 2011, 106 of which were urban and 296 rural), as well as diagnoses of individuals who consulted an authorized physician at least once in each year. Diagnoses are coded according to the German modification of the 10th edition of International Classification of Diseases and Related Health Problems (ICD-10-GM, code J45).^15^

### Definition of asthma cases

We defined a prevalent case of asthma as one diagnosed in at least two quarters of the corresponding year.^16^^–^^18^ In addition, we only included confirmed diagnoses (i.e. those highlighted with the additional diagnostic modifier “assured”). As a sensitivity analysis we also estimated the prevalence based on a single diagnosis of asthma for comparison. An incident case of asthma was defined if it was diagnosed for the first time between 2011 and 2016. Individuals diagnosed with asthma for the first time in 2009 and 2010 were excluded from this analysis.

### Statistical analysis

We calculated the sex- and age-specific prevalence of asthma by dividing the number of asthma diagnoses per sex/age category by the total number of individuals with statutory health insurance in that category, for each separate year from 2009 to 2016. We also calculated the age-standardized prevalence, using the direct standardization method, and 95% confidence intervals (CI), although we note that CIs are not particularly informative because of the very large sample size (> 70 million). As a standard population, we used the German population from the year 2015.^19^ Using the sex- and age-specific prevalence calculations along with sex- and age-specific population distribution, we estimated the total number of individuals affected by asthma in Germany in 2016. 

We performed a spatial analysis of crude asthma prevalence according to administrative district by calculating Global and Local Moran’s *I*.^20^ Districts were divided into four types of areas: (i) rural areas with low population density, that is, a population density lower than 100 inhabitants per km^2^; (ii) rural areas with population concentrations, that is a population density less than 150 inhabitants per km^2^; (iii) urban districts, that is a population density over 150 inhabitants per km^2^; and (iv) large urban municipalities, that is a population above 100 000 inhabitants.^21^ We used semi-parametric group-based modelling of age-standardized prevalence to identify administrative districts with similar trends in prevalence over the study period (2009–2016), that is, longitudinal clusters or trajectories.^22^

Finally, we performed a Kaplan–Meier analysis to estimate the overall summed incidence of asthma as well as by sex, age (children versus adults), type of residence and federal state. We then used a parametric mixed-effect survival model with individuals (level 1) nested within the 402 districts (level 2) to examine the risk of the incidence of asthma according to the abovementioned control variables, with 95% CI. Since the assumption of proportional hazards was violated by an interaction between sex and age, that is, the sex-specific prevalence is not independent of age, and vice versa, we repeated the survival analysis separately by sex to exclude the interactive effects.

## Results

### Study population

The study population of individuals with statutory health insurance with at least one ambulatory health service contact per year comprised 85.33% (70 416 019/82 521 653) of the total German population in 2016 (Table 1). The study sample consisted of 12 169 720 children (0–18 years) and 58 246 299 adults. There were only minor differences across population distributions by age, type of residence and federal state; however, the proportion of females was higher in the study population than in the general population. 

**Table 1:** Demographic characteristics of the study population compared with the general population in asthma morbidity study, Germany, 2016

Characteristic	No. (%)	
	Study population (*n* = 70 416 019)	General population^a^ (*n* = 82 521 653)
**Sex**		
Male	32 084 893 (45.56)	40 697 118 (49.32)
Female	38 331 126 (54.44)	41 824 535 (50.68)
**Age, years**		
0–4	3 448 313 (4.90)	3 756 446 (4.55)
5–9	3 047 833 (4.33)	3 613 927 (4.38)
10–14	2 959 581 (4.20)	3 678 195 (4.46)
15–19	3 468 684 (4.93)	4 172 869 (5.06)
20–24	3 863 057 (5.49)	4 574 031 (5.54)
25–29	4 625 031 (6.57)	5 366 756 (6.50)
30–34	4 455 886 (6.33)	5 221 075 (6.33)
35–39	4 241 230 (6.02)	5 058 038 (6.13)
40–44	3 901 754 (5.54)	4 821 986 (5.84)
45–49	5 034 735 (7.15)	6 259 912 (7.59)
50–54	5 741 805 (8.15)	6 984 307 (8.46)
55–59	5 174 370 (7.35)	6 223 126 (7.54)
60–64	4 396 917 (6.24)	5 281 280 (6.40)
65–69	3 846 002 (5.46)	4 563 301 (5.53)
70–74	3 173 507 (4.51)	3 654 937 (4.43)
≥ 75	9 037 314 (12.83)	9 291 467 (11.26)
**Type of residence^b^**		
Rural areas with low population density	10 219 972 (14.51)	11 857 274 (14.37)
Rural areas with population concentrations	12 297 829 (17.46)	14 028 047 (17.00)
Urban districts	27 732 448 (39.38)	32 400 372 (39.26)
Large urban municipalities	20 165 770 (28.64)	24 235 960 (29.37)
**Federal state**		
Baden-Württemberg	8 944 264 (12.70)	10 951 893 (13.27)
Bavaria	10 742 300 (15.26)	12 930 751 (15.67)
Berlin	3 005 218 (4.27)	3 574 830 (4.33)
Brandenburg	2 167 116 (3.08)	2 494 648 (3.02)
Bremen	597 995 (0.85)	678 753 (0.82)
Hamburg	1 552 606 (2.20)	1 810 438 (2.19)
Hesse	5 264 256 (7.48)	6 213 088 (7.53)
Mecklenburg–Western Pomerania	1 438 593 (2.04)	1 610 674 (1.95)
Lower Saxony	6 884 645 (9.78)	7 945 685 (9.63)
North Rhine–Westphalia	15 547 745 (22.08)	17 890 100 (21.68)
Rhineland–Palatinate	3 358 821 (4.77)	4 066 053 (4.93)
Saarland	856 620 (1.22)	996 651 (1.21)
Saxony	3 648 621 (5.18)	4 081 783 (4.95)
Saxony–Anhalt	2 020 774 (2.87)	2 236 252 (2.71)
Schleswig–Holstein	2 445 762 (3.47)	2 881 926 (3.49)
Thuringia	1 940 683 (2.76)	2 158 128 (2.62)

^a^ German population data for the year 2016 were obtained from the Federal Statistical Office.^19^

^b^ Rural areas with a population density lower than 100 inhabitants per km^2^ were categorized as low population density, rural areas with a population density less than 150 inhabitants per km^2^ were categorized as rural areas with population concentrations, urban districts were defined as districts with a population density over 150 inhabitants per km^2^ and large urban municipalities had a population above 100 000 inhabitants.^21^

### Prevalence

Of the 70 416 019 study individuals, 5 360 867 (7.61%) had at least one diagnosis of asthma. The number of children and adults with at least one diagnosis of asthma was 955 628 (7.85%) and 4 405 239 (7.56%), respectively. Of the study individuals, 4 033 521 had prevalent asthma, classified according to the applied case definition (i.e. two diagnoses), corresponding to a crude prevalence of 5.73%. The crude prevalence among children was 5.13% (624 899/12 169 720) and for adults 5.85% (3 408 622/58 246 299; Table 2). The age-standardized prevalence of asthma was 5.76% (95% CI: 5.76–5.77) in 2016 (Table 3). 

**Table 2:** Temporal trends in asthma prevalence according to sex, age and type of residence, Germany, 2009–2016

Group	Group-specific no. with asthma/Group-specific population (%)							
	2009 (*n* = 70 388 055)	2010 (*n* = 69 073 616)	2011 (*n* = 69 030 407)	2012 (*n* = 68 954 969)	2013 (*n* = 69 700 682)	2014 (*n* = 69 650 700)	2015 (*n* = 69 799 319)	2016 (*n* = 70 416 019)
**Sex**								
Male	1 356 896/ 31 448 561 (4.31)	1 376 692/30 781 881 (4.47)	1 426 721/ 30 863 616 (4.62)	1 444 309/ 30 867 270 (4.68)	1 518 497/ 31 413 253 (4.83)	1 597 817/ 31 418 936 (5.09)	1 642 826/ 31 642 822 (5.19)	1 697 293/ 32 084 893 (5.29)
Female	1 765 481/ 38 939 494 (4.53)	1 823 593/ 38 291 735 (4.76)	1 914 532/ 38 166 791 (5.02)	1 968 252/ 38 087 699 (5.17)	2 064 932/ 38 287 429 (5.39)	2 177 061/ 38 231 764 (5.69)	2 253 437/ 38 156 497 (5.91)	2 336 228/ 38 331 126 (6.09)
**Age, years**								
0–18	627 284/ 12 801 246 (4.90)	627 488/ 12 299 699 (5.10)	620 798/ 12 136 769 (5.12)	603 386/ 11 974 202 (5.04)	611 552/ 11 979 375 (5.11)	635 739/ 11 950 393 (5.32)	631 519/ 11 979 263 (5.27)	624 899/ 12 169 720 (5.13)
> 18	2 495 093/ 57 586 809 (4.33)	2572 797/ 56 773 917 (4.53)	2 720 455/ 56 893 638 (4.78)	2 809 175/ 56 980 767 (4.93)	2 971 877/ 57 721 307 (5.15)	3 139 139/ 57 700 307 (5.44)	3 264 744/ 57 820 056 (5.65)	3 408 622/ 58 246 299 (5.85)
**Type of residence^a^**								
Rural areas with low population density	441 120/ 10 519 783 (4.19)	450 188/ 10 265 569 (4.39)	478 734/ 10 247 756 (4.67)	491 579/ 10 182 055 (4.83)	513 921/ 10 234 622 (5.02)	541 934/ 10 166 646 (5.33)	559 657/ 10 154 938 (5.51)	576 997/ 10 219 972 (5.65)
Rural areas with population concentrations	522 050/ 12 540 333 (4.16)	529 069/ 12 234 886 (4.32)	562 007/ 12 244 041 (4.59)	575 173/ 12 186 818 (4.72)	600 394/ 12 276 268 (4.89)	634 482/ 12 225 503 (5.19)	655 615/ 12 225 091 (5.36)	677 635/ 12 297 829 (5.51)
Urban districts	1 253 114/ 27 937 315 (4.49)	1 280 090/ 27 397 247 (4.67)	1 322 998/ 27 312 724 (4.84)	1 346 288/ 27 274 134 (4.94)	1 411 390/ 27 528 692 (5.13)	1 485 959/ 27 511 326 (5.40)	1 529 994/ 27 531 819 (5.56)	1 582 203/ 27 732 448 (5.71)
Large urban municipalities	906 093/ 19 390 624 (4.67)	940 938/ 19 175 914 (4.91)	977 514/ 19 225 886 (5.08)	999 521/ 19 311 962 (5.18)	1 057 724/ 19 661 100 (5.38)	1 112 503/ 19 747 225 (5.63)	1 150 997/ 19 887 471 (5.79)	1 196 686/ 20 165 770 (5.93)

^a^ Rural areas with a population density lower than 100 inhabitants per km^2^ were categorized as low population density, rural areas with a population density less than 150 inhabitants per km^2^ were categorized as rural areas with population concentrations, urban districts were defined as districts with a population density over 150 inhabitants per km^2^ and large urban municipalities had a population above 100 000 inhabitants.^21^

**Table 3:** Temporal change in age-standardized prevalence of asthma, Germany, 2009–2016

Year	Age-standardized prevalence, % (95 % CI)
2009	4.46 (4.46–4.46)
2010	4.66 (4.66–4.67)
2011	4.87 (4.86–4.87)
2012	4.97 (4.97–4.98)
2013	5.17 (5.16–5.17)
2014	5.45 (5.45–5.46)
2015	5.62 (5.61–5.62)
2016	5.76 (5.76–5.77)

CI: confidence interval.

**Fig. 1:**
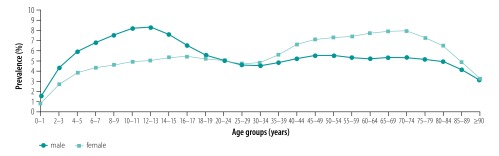
Prevalence of asthma, by sex and age, Germany, 2016

We observed an interaction in terms of sex and age that has already been reported in the literature (Fig. 1).^23^^,^^24^ Asthma prevalence was substantially higher among boys, an association which disappeared in young adulthood. In middle adulthood this association was observed to reverse and asthma prevalence was higher among women, reaching its peak in the age group 65–75 years. After decreasing in boys from the age of 10–11 years until age 30–34 years, there was a slight increase in prevalence in men until age 40–45 years.

From age-specific prevalence data and population distribution data, we estimated that 793 112 children (defined as 0–19 years in this case because of age groups used in the available data) and 3 918 993 adults (> 19 years) were affected by asthma in 2016, resulting in a total of 4 712 106 asthmatics (Table 4).

**Table 4:** Estimated number of asthmatic individuals according to age-specific prevalence and population distribution in Germany, 2016

Age (years)	Men						Women						Estimated total no. with asthma
	Study population			General population			Study population			General population			
	No. in age group	No. with asthma	Age-specific prevalence, %	No. in age group^a^	Estimated no. with asthma		No. in age group	No. with asthma	Age-specific prevalence, %	No. in age group^a^	Estimated no. with asthma		
0–4	1 771 042	59 148	3.34	1 928 588	64 410		1 677 271	34 576	2.06	1 827 858	37 680		102 090
5–9	1 569 396	109 111	6.95	1 857 190	129 120		1 478 437	64 158	4.34	1 756 737	76 235		205 355
10–14	1 515 903	124 047	8.18	1 893 522	154 948		1 443 678	72 759	5.04	1 784 673	89 945		244 892
15–19	1 730 793	107 740	6.22	2 187 398	136 163		1 737 891	91 789	5.28	1 985 471	104 865		241 028
20–24	1 821 178	90 926	4.99	2 395 930	119 622		2 041 879	101 115	4.95	2 178 101	107 861		227 482
25–29	2 100 612	96 094	4.57	2 787 105	127 498		2 524 419	118 397	4.69	2 579 651	120 987		248 486
30–34	2 013 412	91 524	4.55	2 676 180	121 652		2 442 474	116 888	4.79	2 544 895	121 789		243 441
35–39	1 904 544	91 847	4.82	2 557 606	123 341		2 336 686	129 876	5.56	2 500 432	138 977		262 318
40–44	1 747 299	91 723	5.25	2 428 357	127 475		2 154 455	141 423	6.56	2 393 629	157 123		284 597
45–49	2 264 863	124 756	5.51	3 162 743	174 214		2 769 872	196 366	7.09	3 097 169	219 569		393 783
50–54	2 614 771	143 622	5.49	3 526 252	193 687		3 127 034	228 242	7.30	3 458 055	252 403		446 090
55–59	2 373 283	125 996	5.31	3 104 747	164 829		2 801 087	207 805	7.42	3 118 379	231 344		396 173
60–64	2 007 021	105 276	5.25	2 573 457	134 988		2 389 896	184 920	7.74	2 707 823	209 520		344 508
65–69	1 735 885	92 659	5.34	2 186 608	116 718		2 110 117	167 593	7.94	2 376 693	188 765		305 483
70–74	1 408 139	74 983	5.32	1 703 714	90 722		1 765 368	140 265	7.95	1 951 223	155 032		245 754
> 74	3 506 752	167 832	4.79	3 727 721	178 408		5 530 562	340 056	6.15	5 563 746	342 096		520 504
**Total**	**32 084 893**	**1 697 284**	**NA**	**40 697 118**	**2 157 793**		**38 331 126**	**2 336 228**	**NA**	**41 824 535**	**2 554 193**		**4 711 986**

NA: not applicable.

^a^ German population data for the year 2016 were obtained from the Federal Statistical Office.^19^

### Temporal trends

**Fig. 2:**
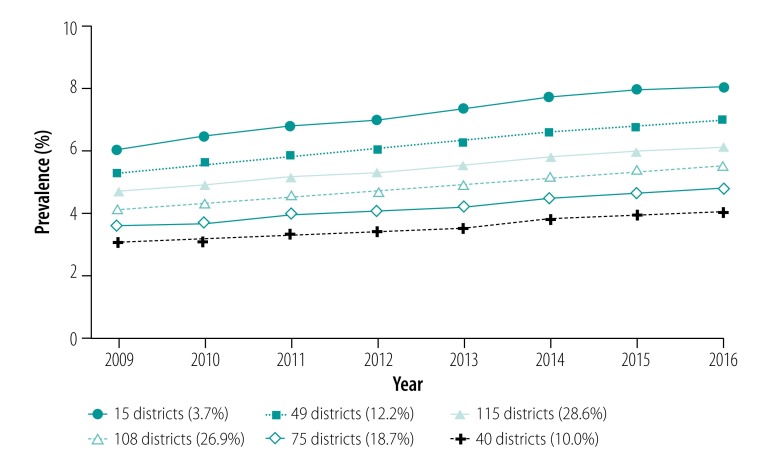
Temporal change in age-standardized asthma prevalence in 402 districts, clustered into six trajectories, Germany, 2009–2016

Notes: We created trajectories using the SAS procedure Proc Traj,^22^ the optimal number of six, which was based on the Bayesian Information Criterion. The German population from the year 2015 was used as a standard population;^19^ the standardization controls for possible demographic changes in the population structure in the study period.

The age-standardized prevalence of asthma increased from 4.46% (95% CI: 4.46–4.46) in 2009 to 5.76% (95% CI: 5.76–5.77) in 2016 (Table 3), corresponding to a relative change of +30.32%. The semi-parametric group-based modelling demonstrated the presence of six clusters of different size, but with a similar course of prevalence over the study period (Fig. 2). We observed an almost linear and relatively uniform increase in prevalence in all clusters from 2009 to 2016. A stratified analysis by age (children versus adults, Table 2) showed that the prevalence increase was attributable to adults (relative change, +35.10%) and the prevalence among children changed only marginally over time (+4.69%).

### Spatial variation

**Fig. 3:**
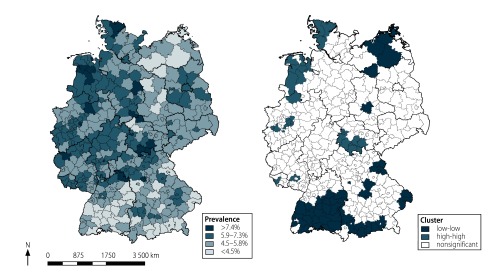
Regional variations in prevalence of asthma and significant spatial clusters, Germany, 2016

Notes: Cartographic presentation of age-standardized prevalence estimates by district (*n* = 402). Equidistant distance was used for group classification. General German population from the year 2015 was used as a standard population,^19^ controlling for possible demographic changes in the population structure in the study period. Districts with significant spatial clusters estimated with Local Moran’s *I*.^20^ Spatial analysis was performed with crude prevalence values.

We observed strong variations by a factor of three over the 402 districts in the age-standardized prevalence of asthma; the lowest and highest prevalences of 3.03% (95% CI: 2.94–3.11) and 8.85% (95% CI: 8.57–9.14) were observed in Schwäbisch Hall and Eisenach, respectively. The age-standardized prevalence was higher in the western parts of Germany and lower in South and East Germany (Fig. 3). We found evidence of a strong spatial autocorrelation at the district level (Global Moran’s *I*: 0.50; *P* < 0.0001). Local Moran’s *I* showed the presence of spatial clusters with high or low prevalence (Fig. 3). Clusters with high prevalence were found in western Lower Saxony, North Rhine–Westphalia, Schleswig–Holstein and in southern Thuringia.

### Incidence

**Fig. 4:**
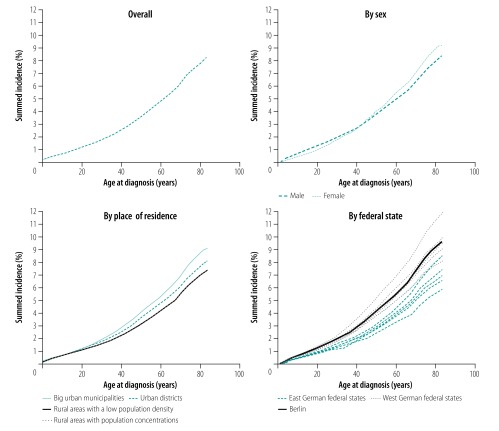
Summed incidence of asthma overall and by sex, type of residence and location as a function of age at diagnosis, Germany, 2009–2016

Notes: Incidence was estimated with Kaplan–Meier analysis. Incidence for each group in Table 4 was added to that of the previous group. In the graph for place of residence, data for rural areas with population concentrations and rural areas with a low population density are superimposed.

The study subpopulation for incidence estimation included 59 289 010 individuals who were included within the statutory health insurance system for several years (not always the full 8-year study period) and who contributed 422 516 235 person-years over the study period. Of this subpopulation, 2 613 755 (4.41%) were categorized as incident asthma cases. The summed incidence was observed to increase almost linearly with increasing age at diagnosis (Fig. 4). The overall incidence rate was 6.19 per 1000 person-years, and was higher among children and adolescents (10.29 per 1000 person-years; 602 264 cases contributing 58 557 060 person-years) than adults (5.53 per 1000 person-years; 2 011 491 cases contributing 363 959 175 person-years). We observed an interaction between sex and age (Fig. 4). We also observed a distinct difference in summed incidence of asthma according to type of residence; the highest incidence proportion was calculated for densely populated large urban municipalities followed by less densely populated urban districts, and the lowest was calculated for rural areas (Fig. 4). The summed incidence was higher in West German compared with East German federal states (Fig. 4).

In multivariable analysis, children had a hazard ratio (HR) of being diagnosed with asthma of 2.17 (95% CI: 2.16–21.8) relative to adults (Table 5). Resident in the western (versus eastern) federal states and resident in densely populated large urban municipalities (versus rural area) were independently associated with an increased risk of asthma, with HRs of 1.33 (95% CI: 1.32–1.34) and 1.32 (95% CI: 1.31–1.32), respectively. The risk of asthma among boys (0–18 years) was over twice as high as that for men (HR: 2.23; 95% CI: 2.22–2.24); we also observed this difference in risk, although not as pronounced, for girls (0–18 years) versus women (HR: 1.59; 95% CI: 1.58–1.59). We also observed sex-specific differences in risk of asthma according to location of residence. Specifically, males living in West Germany had an increased risk of asthma of 1.21 (95% CI: 1.16–1.26) compared with males living in East Germany; this increased risk was only 1.13 (95% CI: 1.08–1.19) for females.

**Table 5:** Crude and adjusted hazard ratios and corresponding 95% confidence intervals for asthma incidence, Germany, 2009–2016

Variables	Crude HR (95% CI)^a^		Adjusted HR (95% CI)^b^		
	Total		Total	Men	Women
**Sex**					
Female	1.01 (1.00–1.01)		1.14 (1.13–1.14)	NA	NA
Male	Reference		Reference	NA	NA
**Age, years**					
0–18	2.17 (2.16–2.18)		1.87 (1.86–1.87)	2.23 (2.22–2.24)	1.59 (1.58–1.59)
> 18	Reference		Reference	Reference	Reference
**East versus West Germany**					
Berlin	1.30 (1.29–1.30)		1.05 (0.75–1.47)	1.05 (0.76–1.44)	1.05 (0.74–1.50)
West Germany	1.33 (1.32–1.34)		1.16 (1.11–1.22)	1.21 (1.16–1.26)	1.13 (1.08–1.19)
East Germany	Reference		Reference	Reference	Reference
**Type of residence^c^**					
Rural areas with a low population density	Reference		Reference	Reference	Reference
Rural areas with population concentrations	1.01 (1.00–1.01)		1.00 (0.95–1.05)	1.01 (0.96–1.05)	1.00 (0.95–1.05)
Urban districts	1.12 (1.11–1.12)		1.03 (0.98–1.08)	1.03 (0.98–1.07)	1.03 (0.98–1.08)
Large urban municipalities	1.32 (1.31–1.32)		1.20 (1.14–1.27)	1.19 (1.13–1.25)	1.22 (1.15–1.29)

CI: confidence interval; HR: hazard ratio; NA: not applicable.

^a^ Cox proportional hazard model.

^b^ Parametric survival model with individuals on level 1 nested within 402 districts on level 2.

^c^ Rural areas with a population density lower than 100 inhabitants per km^2^ were categorized as low population density, rural areas with a population density less than 150 inhabitants per km^2^ were categorized as rural areas with population concentrations, urban districts were defined as districts with a population density over 150 inhabitants per km^2^ and large urban municipalities had a population above 100 000 inhabitants.^21^

## Discussion

In contrast to other studies^25^ that reported a higher prevalence of asthma among children than adults, we estimated relatively similar prevalence values in these two groups. As part of a sensitivity analysis we also assessed the prevalence based on a single diagnosis of asthma, yielding similar prevalence values among children and adults. The wide variations in previously published prevalence estimates,^12^ especially among children, may be explained by methodological differences in those studies, for example, in study design (primary data collection^26^ versus secondary analysis of existing data)^16^ and case definitions (physician diagnosis^16^ versus treated asthma^27^ versus self-reports).^28^ Prevalence estimates and their temporal trends therefore cannot be directly compared between studies.

According to the European Respiratory Society,^29^ an estimated 4 million individuals were affected by asthma in Germany. However, it is not known which study or even which year this estimate is based on, highlighting the scarcity of data. Using the nationwide claims data, we estimated that around 4.7 million individuals in Germany (1 in every 19 children and 1 in every 17 adults) were affected by asthma in 2016. Compared with an estimated number of asthmatics of 3.6 million in 2009 (calculated in the same way), the 2016 figure indicates an increase of over 1 million individuals. This increase is mostly attributable to an increase in adults with asthma; in contrast, asthma prevalence seems to have stabilized among children over our study period. Our observed stability in childhood asthma prevalence is in agreement with other studies in Germany;^30^^,^^31^ one study observed only minor changes in prevalence between 2006–2009 (3.7%) and 2014–2017 (4.0%).^30^

Research on the incidence rate of asthma in Germany is scarce.^32^^,^^33^ A prospective study with follow-up visits between 1992 and 2005 among a relatively small sample of about 3200 children from three counties in East Germany yielded an incidence rate of 5.0 per 1000 person-years.^32^ In an analysis of around 4000 adult participants of the nationwide German Health Interview and Examination Survey for Adults, an incidence rate for asthma over an average period of 12 years (1997–2011) of 1.1–3.4 per 1000 person-years was observed.^33^ We estimated higher incidence rates (10.3 and 5.5 per 1000 person-years for children and adults, respectively); however, both studies mentioned above were regionally restricted and had small sample sizes. Our estimates are in good agreement with findings from large-scale studies in other high-income countries, including Canada (10.9 and 5.6 per 1000 person-years for children aged 5–9 years and 10–14 years, respectively, and 2.8 for adults aged 40–69 years),^34^ the United Kingdom (4.1 and 9.9 per 1000 person-years for adults and children, respectively)^35^ and the USA (3.8 and 12.5 per 1000 person-years for adults and children, respectively).^36^

Regarding the regional distribution of asthma morbidity, we observed several differences in terms of rural versus urban areas, East versus West German federal states and small district-scale hotspots. First, we observed differences in asthma morbidity between those resident in rural and urban areas. Although a higher morbidity of asthma in urban versus rural areas has previously been observed,^7^ already partly explained by the hygiene hypothesis,^9^ we also differentiated rural and urban districts by population density. We found higher incidence rates in densely populated large urban municipalities than in lower-population urban districts; environmental factors such as air pollution, one of the key risk factors for asthma development,^37^ may explain this association. 

Lower asthma morbidity was observed for residents of East Germany compared with those of West Germany at the beginning of 1990, shortly after the reunification of Germany.^13^ Data from 2003–2006 did not show any difference, implying that morbidity had increased in East Germany;^26^ however, the authors of that study suggested that unmeasured confounding factors may have masked a regional difference. Our observed regional difference is supported by a study that compared asthma prevalence in children with a similar genetic ancestry but living in different environments (in our case, genetically similar German children living in West or East Germany).^38^ For example, a study measuring the asthma prevalence among Chinese children, showed that the prevalence increased from those born in China to those who migrated to Canada, and was highest for those born in Canada.^38^ Epidemiological studies that rely on objectively measured data (e.g. skin prick test) also support our findings; for example, a higher atopic sensitization rate in children from West compared with those from East Germany has been reported.^13^

We also observed district-scale variations in prevalence of asthma, and identified hot and cold spots, that is, districts with high and low asthma prevalence estimates, respectively. The distribution of local environmental risk factors of asthma (e.g. allergens, prenatal smoking, nutrition and/or stress) across the districts is usually unknown, but there is some evidence for district-level variations in smoking among men and women.^39^ Other factors contributing to regional variations in asthma morbidity include meteorological factors (e.g. solar radiation),^40^ area-level socioeconomic status, and the different diagnostic coding behaviour and practices of physicians. We found distinct differences between different coastal areas; specifically, we observed a cluster of high-prevalence districts near the North Sea coast, but a low-prevalence cluster in the Baltic Sea coastal region, a finding which merits further investigation.

Our study benefited from the use of nationwide claims data, incorporating outpatient diagnoses of approximately 85% of the German population. As there were only minor differences between the study population and the general German population in terms of age, type of residence and federal state, our findings may be considered representative. Our spatial analysis at the district level, allowing the identification of high-risk areas, is also important as there is no consensus on primary prevention of asthma. 

Our study had several limitations. Our study population may not be representative in terms of sex distribution, as the proportion of females in our study population was slightly higher than that in the general population. The 15% of the population that is privately insured, whose data are not included, may differ from the individuals with statutory health insurance in terms of socioeconomic status. Differences in morbidity between individuals with private or statutory health insurance in Germany have been shown,^41^ although not explicitly for asthma. One study does mention an asthma prevalence (around 5%) among privately insured individuals in Germany, comparable to our estimated prevalence for the entire population, but this estimate was based on a personal communication.^27^ Also, physician claims data are primarily collected for billing purposes and not for morbidity research. Although we cannot rule out a degree of misdiagnosis, our conservative case definition (a diagnosis of asthma in at least two quarters of the year in question) reduced the possibility of counting false-positive cases. This case definition was also used in other studies of asthma prevalence, including in China, Taiwan^42^ (which required diagnoses in at least three quarters of each year), Germany,^16^ the Republic of Korea^17^ and the USA.^18^ Finally, information on potential confounding factors, such as smoking and socioeconomic status, was not available from the physician claims data.

We conclude that asthma is a common disorder in Germany with an increasing disease burden. A recent estimate of a current annual cost of asthma treatment per patient of €2168 Euros (€)^43^ applied to our calculated number of prevalent asthma cases in 2016 (> 4 million) results in a cost to the German health-care system of over €8 billion. To control this increasing asthma prevalence and its associated costs, more research into the prevention and causes of asthma is required. We anticipate that our insights into the spatial distribution of asthma morbidity will serve as a solid basis for public health interventions, including region-specific prevention programmes and control.

## References

[1] The global asthma report 2018. Auckland: The Global Asthma Network; 2018. Available from: www.globalasthmanetwork.org [cited 2019 Sep 17].

[2] Masoli M, Fabian D, Holt S, Beasley R; Global Initiative for Asthma (GINA) Program. The global burden of asthma: executive summary of the GINA Dissemination Committee report. Allergy. 2004 5;59(5):469–78. 10.1111/j.1398-9995.2004.00526.x 15080825

[3] To T, Stanojevic S, Moores G, Gershon AS, Bateman ED, Cruz AA, et al. Global asthma prevalence in adults: findings from the cross-sectional world health survey. BMC Public Health. 2012 3 19;12(1):204. 10.1186/1471-2458-12-204 22429515 PMC3353191

[4] Krstić G. Asthma prevalence associated with geographical latitude and regional insolation in the United States of America and Australia. PLoS One. 2011 4 8;6(4):e18492. 10.1371/journal.pone.0018492 21494627 PMC3072993

[5] Gupta RP, Mukherjee M, Sheikh A, Strachan DP. Persistent variations in national asthma mortality, hospital admissions and prevalence by socioeconomic status and region in England. Thorax. 2018 8;73(8):706–12. 10.1136/thoraxjnl-2017-210714 30006496 PMC6204968

[6] Mallol J, Solé D, Baeza-Bacab M, Aguirre-Camposano V, Soto-Quiros M, Baena-Cagnani C; The Latin American ISAAC Group. Regional variation in asthma symptom prevalence in Latin American children. J Asthma. 2010 8;47(6):644–50. 10.3109/02770901003686480 20642377

[7] Timm S, Frydenberg M, Janson C, Campbell B, Forsberg B, Gislason T, et al. The urban-rural gradient in asthma: a population-based study in Northern Europe. Int J Environ Res Public Health. 2015 12 30;13(1):93. 10.3390/ijerph13010093 26729146 PMC4730484

[8] Gern JE. The urban environment and childhood asthma study. J Allergy Clin Immunol. 2010 3;125(3):545–9. 10.1016/j.jaci.2010.01.037 20226291 PMC2860857

[9] Strachan DP. Hay fever, hygiene, and household size. BMJ. 1989 11 18;299(6710):1259–60. 10.1136/bmj.299.6710.1259 2513902 PMC1838109

[10] Ege MJ, Mayer M, Normand AC, Genuneit J, Cookson WO, Braun-Fahrländer C, et al.; GABRIELA Transregio 22 Study Group. Exposure to environmental microorganisms and childhood asthma. N Engl J Med. 2011 2 24;364(8):701–9. 10.1056/NEJMoa1007302 21345099

[11] Braun-Fahrländer C, Riedler J, Herz U, Eder W, Waser M, Grize L, et al.; Allergy and Endotoxin Study Team. Environmental exposure to endotoxin and its relation to asthma in school-age children. N Engl J Med. 2002 9 19;347(12):869–77. 10.1056/NEJMoa020057 12239255

[12] Aumann I, Prenzler A, Welte T, Gillissen A. [Epidemiology and costs of asthma in Germany - a systematic literature review]. Pneumologie. 2014 8;68(8):557–67. German. 25003905 10.1055/s-0034-1377225

[13] von Mutius E, Martinez FD, Fritzsch C, Nicolai T, Roell G, Thiemann HH. Prevalence of asthma and atopy in two areas of West and East Germany. Am J Respir Crit Care Med. 1994 2;149(2):358–64. 10.1164/ajrccm.149.2.8306030 8306030

[14] Steppuhn H, Kuhnert R, Scheidt-Nave C. 12-month-prevalence of asthma among adults in Germany. J Health Mon. 2017;2(3):36–45. 10.17886/RKI-GBE-2017-064 PMC10165913 37168951

[15] ICD-10-GM 2019 [internet]. Cologne: Deutsches Institut für Medizinische Dokumentation und Information; 2019. German. Available from: https://www.dimdi.de/dynamic/de/klassifikationen/icd/icd-10-gm/ [cited 2019 Oct 19].

[16] Hasford J, Uricher J, Tauscher M, Bramlage P, Virchow JC. Persistence with asthma treatment is low in Germany especially for controller medication - a population based study of 483,051 patients. Allergy. 2010 3;65(3):347–54. 10.1111/j.1398-9995.2009.02161.x 19712117

[17] Shin JY, Sohn KH, Shin JE, Park M, Lim J, Lee JY, et al. Changing patterns of adult asthma incidence: results from the National Health Insurance Service-National Sample Cohort (NHIS-NSC) database in Korea. Sci Rep. 2018 10 9;8(1):15052. 10.1038/s41598-018-33316-y 30302007 PMC6177405

[18] Gray CL, Lobdell DT, Rappazzo KM, Jian Y, Jagai JS, Messer LC, et al. Associations between environmental quality and adult asthma prevalence in medical claims data. Environ Res. 2018 10;166:529–36. 10.1016/j.envres.2018.06.020 29957506 PMC6110955

[19] Statistisches Bundesamt [internet]. Wiesbaden: Statistisches Bundesamt (Destatis); 2019. Available from: www.destatis.de [cited 2019 Sep 17].

[20] Anselin L. Local indicators of spatial association - LISA. Geogr Anal. 1995;27(2):93–115. 10.1111/j.1538-4632.1995.tb00338.x

[21] Bundesinstitut für Bau-, Stadt- und Raumforschung [internet]. Bonn: Bundesamt für Bauwesen und Raumordnung; 2019.German. Available from: https://www.bbsr.bund.de/BBSR/DE/Bundesinstitut/bundesinstitut_node.html [cited 2019 Oct 2019].

[22] Jones B, Nagin D, Roeder K. A SAS procedure based on mixture models for estimating developmental trajectories. Sociol Methods Res. 2001;29(3):374–93. 10.1177/0049124101029003005

[23] Arbes S, Guo X, Orelien J, Zeldin D. Interaction between sex and age in the prevalence of current asthma. J Allergy Clin Immunol. 2004;113(2) Suppl:S302 10.1016/j.jaci.2004.01.577

[24] Carey MA, Card JW, Voltz JW, Arbes SJ Jr, Germolec DR, Korach KS, et al. It’s all about sex: gender, lung development and lung disease. Trends Endocrinol Metab. 2007 10;18(8):308–13. 10.1016/j.tem.2007.08.003 17764971 PMC2391086

[25] Dharmage SC, Perret JL, Custovic A. Epidemiology of asthma in children and adults. Front Pediatr. 2019 6 18;7:246. 10.3389/fped.2019.00246 31275909 PMC6591438

[26] Schlaud M, Atzpodien K, Thierfelder W. [Allergic diseases. Results from the German Health Interview and Examination Survey for Children and Adolescents (KiGGS)]. Bundesgesundheitsblatt – Gesundheitsforschung - Gesundheitsschutz. 2007 May-Jun;50(5-6):701–10. German. 10.1007/s00103-007-0231-9 17514454

[27] Stock S, Redaelli M, Luengen M, Wendland G, Civello D, Lauterbach KW. Asthma: prevalence and cost of illness. Eur Respir J. 2005 1;25(1):47–53. 10.1183/09031936.04.00116203 15640322

[28] Genuneit J, Weinmayr G, Radon K, Dressel H, Windstetter D, Rzehak P, et al. Smoking and the incidence of asthma during adolescence: results of a large cohort study in Germany. Thorax. 2006 7;61(7):572–8. 10.1136/thx.2005.051227 16537668 PMC2104663

[29] European Respiratory Society. European Lung White Book. Asthma Burden. Available from: https://www.ersnet.org/images/stories/pdf/asthma.pdf [cited 2019 Oct 20].

[30] Poethko-Müller C, Thamm M, Thamm R. Hay fever and asthma among children and adolescents in Germany: cross-sectional findings from the KiGGS wafe 2 and trends. J Health Mon. 2018;3(1):55–9.

[31] Zöllner IK, Weiland SK, Piechotowski I, Gabrio T, von Mutius E, Link B, et al. No increase in the prevalence of asthma, allergies, and atopic sensitisation among children in Germany: 1992-2001. Thorax. 2005 7;60(7):545–8. 10.1136/thx.2004.029561 15994260 PMC1747445

[32] Rzehak P, Schoefer Y, Wichmann HE, Heinrich J. A prospective study on the association between hay fever among children and incidence of asthma in East Germany. Eur J Epidemiol. 2008;23(1):17–22. 10.1007/s10654-007-9205-3 17985197

[33] Steppuhn H, Buda S, Wienecke A, Kraywinkel K, Tolksdorf K, Haberland J, et al. Time trends in incidence and mortality of respiratory diseases of high public health relevance in Germany. J Health Mon. 2017;2(3):3–33. 10.17886/RKI-GBE-2017-061 PMC10165912 37168954

[34] Gershon AS, Guan J, Wang C, To T. Trends in asthma prevalence and incidence in Ontario, Canada, 1996-2005: a population study. Am J Epidemiol. 2010 9 15;172(6):728–36. 10.1093/aje/kwq189 20716702

[35] Simpson CR, Sheikh A. Trends in the epidemiology of asthma in England: a national study of 333,294 patients. J R Soc Med. 2010 3;103(3):98–106. 10.1258/jrsm.2009.090348 20200181 PMC3072257

[36] Winer RA, Qin X, Harrington T, Moorman J, Zahran H. Asthma incidence among children and adults: findings from the Behavioral Risk Factor Surveillance system asthma call-back survey–United States, 2006-2008. J Asthma. 2012 2;49(1):16–22. 10.3109/02770903.2011.637594 22236442

[37] Nardone A, Neophytou AM, Balmes J, Thakur N. Ambient air pollution and asthma-related outcomes in children of color of the USA: a scoping review of literature published between 2013 and 2017. Curr Allergy Asthma Rep. 2018 4 16;18(5):29. 10.1007/s11882-018-0782-x 29663154 PMC6198325

[38] Wang HY, Wong GW, Chen YZ, Ferguson AC, Greene JM, Ma Y, et al. Prevalence of asthma among Chinese adolescents living in Canada and in China. CMAJ. 2008 11 18;179(11):1133–42. 10.1503/cmaj.071797 19015564 PMC2582762

[39] Kroll LE, Lampert T. [Regionalization of health indicators. Results from the GEDA-Study 2009]. Bundesgesundheitsblatt Gesundheitsforschung Gesundheitsschutz. 2012 1;55(1):129–40. German. 10.1007/s00103-011-1403-1 22286258

[40] Krstić G. Asthma prevalence associated with geographical latitude and regional insolation in the United States of America and Australia. PLoS One. 2011 4 8;6(4):e18492. 10.1371/journal.pone.0018492 21494627 PMC3072993

[41] Huber J, Mielck A. [Morbidity and health care among statutory and privately insured individuals]. Bundesgesundheitsblatt Gesundheitsforschung Gesundheitsschutz. 2010;53:925–38. German. 10.1007/s00103-010-1119-7 20853090

[42] Ma YC, Lin CC, Yang SY, Chen HJ, Li TC, Lin JG. Time trend analysis of the prevalence and incidence of diagnosed asthma and traditional Chinese medicine use among adults in Taiwan from 2000 to 2011: A population-based study. PLoS One. 2015 10 20;10(10):e0140318. 10.1371/journal.pone.0140318 26484761 PMC4618865

[43] Jacob C, Bechtel B, Engel S, Kardos P, Linder R, Braun S, et al. Healthcare costs and resource utilization of asthma in Germany: a claims data analysis. Eur J Health Econ. 2016 3;17(2):195–201. 10.1007/s10198-015-0671-3 25716136 PMC4757601

